# Growing Evidence for LV Unloading in VA ECMO

**DOI:** 10.3390/jcm12186069

**Published:** 2023-09-20

**Authors:** Jan Soltes, Daniel Rob, Petra Kavalkova, Jan Bruthans, Jan Belohlavek

**Affiliations:** 1 Department of Anaesthesiology and Intensive Care Medicine, First Faculty of Medicine, Charles University in Prague and General University Hospital in Prague, 12808 Prague, Czech Republic; 2 Emergency Service of Central Bohemia, 27201 Kladno, Czech Republic; 3 2nd Department of Medicine—Department of Cardiovascular Medicine, First Faculty of Medicine, Charles University in Prague and General University Hospital in Prague, 12808 Prague, Czech Republic

**Keywords:** ECMO, Impella, ECPELLA, ECMELLA, unloading, IABP, cardiogenic shock

## Abstract

Impressively increasing availability of mechanical circulatory/cardiac support systems (MCSs) worldwide, together with the deepening of the knowledge of critical care medical practitioners, has inevitably led to the discussion about further improvements of intensive care associated to MCS. An appealing topic of the left ventricle (LV) overload related to VA ECMO support endangering myocardial recovery is being widely discussed within the scientific community. Unloading of LV leads to the reduction in LV end-diastolic pressure, reduction in pressure in the left atrium, and decrease in the LV thrombus formation risk. Consequently, better conditions for myocardial recovery, with comfortable filling pressures and a better oxygen delivery/demand ratio, are achieved. The combination of VA ECMO and Impella device, also called ECPELLA, seems to be a promising strategy that may bring the improvement of CS mortality rates. The series of presented trials and meta-analyses clearly showed the potential benefits of this strategy. However, the ongoing research has brought a series of new questions, such as whether Impella itself is the only appropriate unloading modality, or any other approach to unload LV would be beneficial in the same way. Benefits and potential risks of LV unloading and its timing are being discussed in this current review.

## 1. Introduction

Widely increased availability of mechanical circulatory/cardiac support systems (MCSs), together with the deepening of the knowledge of critical care medical practitioners, has inevitably led to the discussion about further improvements of intensive care associated to MCS. Cardiogenic shock (CS) is one of the main indications for using MCS, related to not only acute coronary syndromes but also myocarditis, end-stage heart failure, postcardiotomy, and other etiologies. CS is associated with significant hospital mortality [1]. The approach to the treatment of this serious condition has, by virtue of MCS, undergone dramatic changes during the recent decades. A recent randomized controlled trial [2] has shown very controversial effect of using VA ECMO in patients suffering cardiogenic shock from a variety of reasons, mainly acute myocardial infarction. Definitely, careful consideration and appropriate patient selection remain the mainstays of using this rescue option having in mind the considerably deleterious effect of VA ECMO on the primarily dysfunctional left ventricle (LV). Another new multicenter randomized trial [3] and meta-analysis of randomized trials both revealed the results explaining the non-superiority of survival rates in VA ECMO-treated patients when compared with conservative treatment [4]. Thus, innovative ways to improve the outcome of this desperate patient population are being explored. Mainly, the concept of LV unloading leads in this area; therefore, pathophysiology and rising evidence for LV unloading initiation will be discussed in this review.

Since its introduction in 1972 [5], there has been a significant rise [6,7] in the utilization of peripheral VA ECMO for refractory cardiogenic shock, as evidenced by national trends. Since 1990, data from the Extracorporeal Life Support Organization registry indicate that over 15,000 adult patients have received VA ECMO support, with a hospital discharge survival rate of approximately 40%. Various single-center studies have also supported the use of VA ECMO for carefully selected patients with refractory cardiogenic shock. Moreover, many medical centers have explored the potential of peripheral VA ECMO in managing refractory cardiac arrest, a technique known as extracorporeal cardiopulmonary resuscitation (ECPR).

Nevertheless, it is important to note that randomized controlled studies in this context are very challenging primarily due to logistical, legal, and ethical challenges associated with conducting such trials involving patients with cardiac arrest or severe cardiogenic shock. While some indications of improved survival and neurological outcomes have been observed in specific patient subgroups receiving ECPR for refractory cardiac arrest, these benefits are more prominent in cases of in-hospital cardiac arrests (IHCAs) and instances where immediate and effective bystander cardiopulmonary resuscitation is administered, with minimal delays in initiating VA ECMO [8].

Overall, the reported survival rates with peripheral VA ECMO in cardiac arrest and refractory cardiogenic shock typically range between 29% (for extracorporeal cardiopulmonary resuscitation) and 41% (for refractory cardiogenic shock) [9,10]. Due to the absence of robust evidence, current guidelines offer only a low-level recommendation for the use of VA ECMO, and even then, it is primarily suggested for cases of cardiac arrest.

## 2. Terminology

**Scheme 1:**
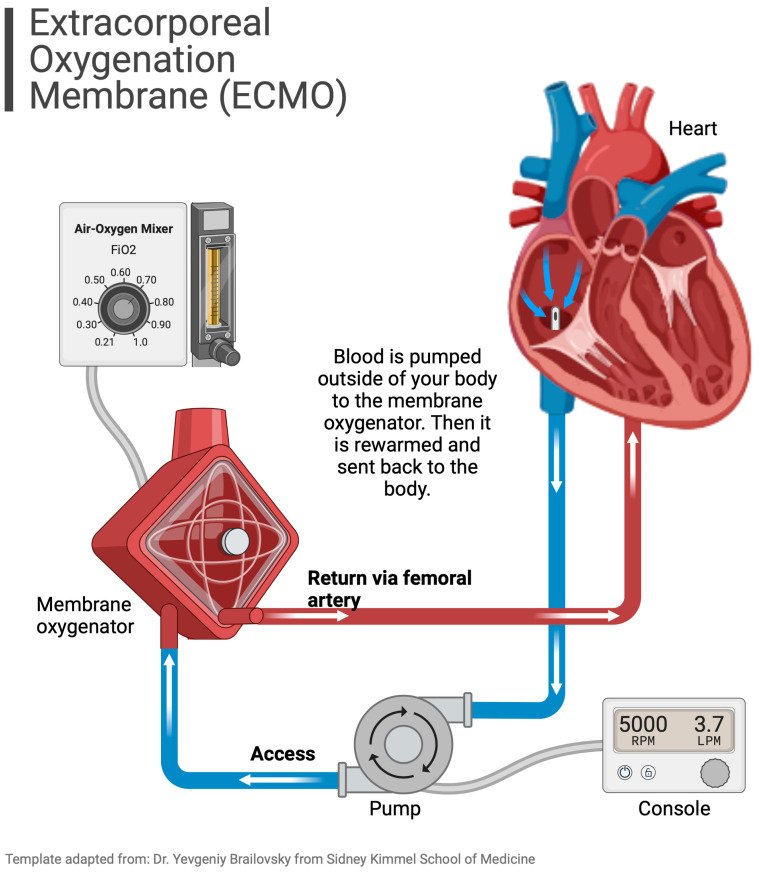
VA ECMO.

Implantation of VA ECMO is followed by a rapid restoration of tissue perfusion; this is obvious from the term mechanical circulatory support. Yet, the term mechanical cardiac support also exists, but it should not be used in the context of VA ECMO, as it provides no augmentation to the LV function. Therefore, it is more appropriate to save this term for left or right ventricular assist devices (LVADs/RVADs), which are actually able to provide support to myocardial function. Atti et al. (2022), in their comprehensive review, described various types and modifications of mechanical circulatory and cardiac support systems. In Table 1, simplified classification is shown. Most of the existing pumps were initially created for LV assistance. Currently, the FDA (Federal Drug Administration) has not granted approval for durable MCS devices specifically designed for right ventricular (RV) support. These devices can be categorized as either short-term/temporary or long-term/durable based on the duration of mechanical support they provide. Additionally, pumps can be classified according to their mechanism of action, falling into categories such as volume displacement pumps, centrifugal pumps, or axial flow pumps [11]. In Scheme 1, a scheme of VA ECMO is shown.

## 3. Applied Physiology

VA ECMO serves as a temporary mechanical circulatory support system with simultaneous external gas exchange for individuals facing acute cardiorespiratory failure. Each VA ECMO setup includes components such as a venous cannula (for inflow and drainage), a centrifugal pump, an oxygenator, and an arterial cannula (for outflow and return). VA ECMO cannulas can be inserted through either peripheral or central access methods. Central VA ECMO configuration can mainly be seen in cardiac surgery theaters to provide short-term assistance, typically for patients with postcardiotomy heart failure who are struggling to be weaned off cardiopulmonary bypass [8].

On the other hand, peripheral VA ECMO can be initiated either percutaneously or through surgical intervention outside an operating room. This is typically reserved for patients experiencing refractory cardiogenic shock and cardiac arrest, with access via the femoral artery and femoral or internal jugular vein. Alternatively, a different approach utilizes standard venous access (either through the femoral or internal jugular vein) with arterial return directed to an ascendent aorta via vascular graft [8]. This configuration represents a kind of hybrid peripheral–central VA ECMO.

A general approach for the implantation of VA ECMO under acute conditions is peripheral, bifemoral (*femoral vein* and *artery* are used) configuration. A venous cannula is inserted though the inferior vena cava directly to the right atrium; the blood is being drawn into the extracorporeal circuit and flows through the complex of an oxygenator and centrifugal pump to enter the return arterial cannula, which expels the blood flow retrogradely through the arterial system into the abdominal and thoracic aorta and provides perfusion to tissues and organs [12,13].

### 3.1. VA ECMO-Generated Circulation and Harlequin Syndrome

Blood flow generated by VA ECMO is non-physiological. The blood stream is directed against the physiological flow (if any is preserved) in the aorta. Thus, two extreme conditions can emerge. If the LV function is maintained to some extent, then there is a meeting (mixing) point of the physiological and ECMO blood stream somewhere in the aortic arch. Thus, if the LV function is preserved enough to shift this mixing point distal from the origin of the *left common carotid artery* (or *left subclavian artery*), a condition called Harlequin syndrome may ensue. Harlequin, or north–south syndrome is reported in 8.8% of cases [14]. Pathophysiologically, the main issue is that the blood from the native output of the LV may be poorly oxygenated due to concomitant pulmonary dysfunction. On the other hand, ECMO-generated blood stream contains highly oxygenated blood. As a result of the phenomenon, hypoxia of areas supplied by the branches originating from the aortic arch and perfused by the native left ventricle output may be observed. This is particularly dangerous considering a possibility of cerebral hypoxic damage [12,15]. Not less importantly, the myocardium itself is compromised in this situation, as coronary arteries originate from the most proximal part of the aorta [13]. Owing to these anatomical considerations, the myocardium may still suffer from inadequate oxygen delivery. Based on explained hemodynamic principles, monitoring via an arterial line for the sampling of blood gases should be placed in the artery of the right upper extremity (typically *radial artery*). Another possibility to detect hypoxic cerebral perfusion is the use of near-infrared-spectroscopy (NIRS) monitoring, attached to patient’s forehead [15]. If pulmonary dysfunction is advanced and the LV function is better than expected, the appropriate solution seems to be adding another returning venous cannula into the *internal jugular vein* and thus upgrading to V-AV ECMO to assure adequate oxygenation of the blood leaving the LV.

### 3.2. Left Ventricle Overload

Thanks to large bore cannulas and modern pumps, VA ECMO can provide substantial flow support; typical flows usually range around 3 to 4.5 L per minute. VA ECMO achieves this by directly drawing blood from the systemic venous system, which reduces the right ventricular preload and alleviates peripheral venous congestion. The flow rate (Q) is determined by the pressure gradient created by the pump and is significantly influenced by the cannula’s radius (directly proportional to r^4). Additionally, it is inversely proportional to fluid viscosity (η) and cannula length (l), as per Poiseuille’s law Q = πPr^4/8ηl [8].

While it may seem that this redirection of blood away from the heart would also reduce the LV preload and alleviate pulmonary congestion, this is not the right understanding of the situation. This is partly due to the fact that ECMO, despite providing higher flows, elevates blood pressure. Consequently, even with the increased ECMO-induced arterial flow, some blood continues to flow through the pulmonary circuit (as not all of it is diverted into the drainage cannula and instead passing through the right atrium and right ventricle). Factors such as the Thebesian drainage of coronary blood flow, the presence of aortic regurgitation, and the return of bronchial blood flow to the LA contribute to blood returning to the LV. To exit the LV, blood must overcome the ECMO-induced increase in arterial pressure, necessitating the LV to generate sufficient pressure [8].

It is obvious that in patients with peripheral VA ECMO, it is not possible to completely drain the blood entering the heart; thus, residual basic circulation through the natural pathway still exists. This phenomenon may cause serious clinical issues in another extreme hemodynamic situation, which will be emphasized in this review. In case of severe LV dysfunction without any effective stroke volume, the impaired function of the LV is furthermore deteriorated by the increase in the afterload caused by the blood stream originating from the ECMO. As a consequence of this, typical hemodynamic pattern may [11,13] be seen:

(1) increased end-diastolic LV pressure (LVEDP);
(2) increased LV wall tension;
(3) worsening of mitral valve regurgitation;
(4) increased LA pressure;
(5) elevation of pulmonary venous pressure;
(6) increased risk of LV thrombosis

Even if there would not be clinically significant deterioration in LV pulsatility, the pathophysiology connected to the adaptation of the LV to the increased afterload is working behind the scenes. Thus, firstly, LVEDP is increased, which is followed with increased calcium sensitivity of cardiomyocytes resulting in higher contractile power. These adaptation mechanisms, important for the preservation of the LV stroke volume despite the increased afterload, are tightly tied to a higher oxygen demand of the myocardium [14].

Adjusting VA ECMO flow to the lowest sufficient value accompanied with thorough restrictive fluid management using diuretics, hemodialysis (CVVHD), or continuous veno-venous hemofiltration (CVVH) and inotrope agents might help with left heart decompression. However, this conservative approach might not be possible in most severe cardiogenic shock patients presented with a completely akinetic LV.

Patients with aortic valve regurgitation and a competent mitral valve are logically more endangered with the LV overload. Considering another valve pathology, the presence of mitral valve regurgitation enables the transmission of pressure from an overloaded LV into the LA and pulmonary circulation (increase in PCWP (pulmonary capillary wedge pressure) and PAP (pulmonary artery pressure) is measured) resulting in the congestion and deterioration of oxygenation.

In *extremis*, when the leaflets of the aortic valve are not being opened, clinically presented as a non-pulsatile flow, the thrombosis of the aortic valve, aortic root, and LV may occur (with following fatal systemic thromboembolic complications). Described alterations in the physiology of the heart may lead not only to the development of pulmonary edema (by the increase in pulmonary venous pressure) but even to the worsening of myocardial ischemia (due to the elevation of LV wall tension and LVEDP) and the frequency of malignant ventricular arrhythmias. These changes can be summarized in terms of increased mechanical stress and strain.

### 3.3. How to Recognize LV Overload?

Detecting LV distention and pulmonary edema during VA ECMO support plays a crucial role in patient care. Various clinical indicators can be utilized to monitor and identify patients who may be at risk. Firstly, one straightforward approach involves assessing the presence and extent of aortic valve opening through arterial pulse pressure tracing. As ECMO flow increases, the mean arterial pressure rises, but the pulse pressure and stroke volume decrease, indicating a reduction in aortic valve opening [8].

Secondly, echocardiography can provide direct visualization of the degree and duration of aortic valve opening. The use of an M-Mode through the aortic valve can help determine if and to what degree the aortic valve is opening. However, it is worth noting that when it comes to assessing LV distention, echocardiography may have limitations. This is because changes in LV dimensions may not accurately reflect changes in LVEDP due to the nonlinear nature of the LVEDP relationship and pericardial constraints [8]. Additionally, variations in the LV chamber size during ECMO support can be misleading as indicators of ventricular distention may be influenced by previous pathology (present before VA ECMO).

Thirdly, progressive hypoxia in blood exiting the LV, which can be measured from the right radial artery or by cerebral oximetry, may indicate the perfusion of the upper circulation with deoxygenated blood due to worsening pulmonary edema [8] Lastly, deteriorating pulmonary edema observed in chest X-rays can signal increasing PCWP. However, this finding may occur late and lacks specificity, as radiographic changes can also result from other conditions such as acute respiratory distress syndrome or infection. While each of these four measures can help detect aortic valve opening and LV loading, they only offer indirect indicators of monitoring for increases in PCWP. The most reliable index for assessing LV filling pressures is to have a pulmonary artery catheter (PAC) in place and directly measure either the pulmonary artery diastolic pressure or PCWP [8]

Unloading of the LV seems to be a reasonable solution to preserve the LV function by decreasing the energy demand and possibly achieve better outcomes. Several modalities were described (Table 2) for this purpose [11,13,16]:

(1) pharmacological management (maintaining of the appropriate stroke volume by the administration of inotropic agents, i.e., dobutamine, PDE III inhibitors, levosimendan);
(2) surgical (vent in the LV/left atrium/pulmonary artery);
(3) percutaneous devices (such as an intra-aortic balloon pump (IABP) and percutaneous axial flow devices like the Impella family by Abiomed).

**Table 2:** Different modalities of LV unloading (based on Belohlavek et al. [12] and Meani et al. [17]; LA = left atrium, PA = pulmonary artery, LV = left ventricle).

Location-Device	Access	Output (Max)
LA-vent	Surgical	Undetermined
LA-vent	Percutaneous, transseptal	Undetermined
Atrial septostomy	Percutaneous	Undetermined
PA-vent	Percutaneous/surgical	Undetermined
LV-vent	Surgical	Undetermined
LV-Impella 2.5/CP	Percutaneous	2.5/3.7 L/min
LV-Impella 5.0/5.5	Surgical	5.0/5.5 L/min
Aorta-IABP	Percutaneous	
LA-TandemHeart	Percutaneous	5 L/min

Considering the surgical approach sternotomy or thoracotomy is usually used to place a venting cannula into the pulmonary vein, directly to the LV, left atrium, or even pulmonary artery. An obvious disadvantage of these methods is the degree of invasiveness and high risk of bleeding complications.

Therefore, percutaneous methods with limited invasiveness have become most widely used. Nowadays, in 84% of cases, LV unloading was provided by percutaneous devices, and 16% of cases underwent surgical procedure in order to unload the overdistended LV [16].

### 3.4. Impella Device—The New Hope for Effective LV Unloading

The Impella device is a catheter-based, small measured percutaneous axial flow LVAD, which is able to create a blood flow of up to 2.5/3.5/5.5 L/min depending on a particular type of the machine. The Impella family currently contains Impella CP, 5.5, and RP (for the right heart support) [18,19]. Impella CP is implanted via the *femoral artery*; Impella 5.5 requires arteriotomy and a vascular graft.

### 3.5. History of Impella

The origin of the idea of how blood flow is created in Impella leads to the ancient Greece, where Archimedes’s screw was invented (approx. 200–300 BC). The American physician Richard Wampler, a father of the reborn idea, developed a predecessor of the Impella in 1985. After years of improvements, in approx. 2000, finally the Impella family of devices was revealed [18,19].

### 3.6. Why to Unload?

**Scheme 2:**
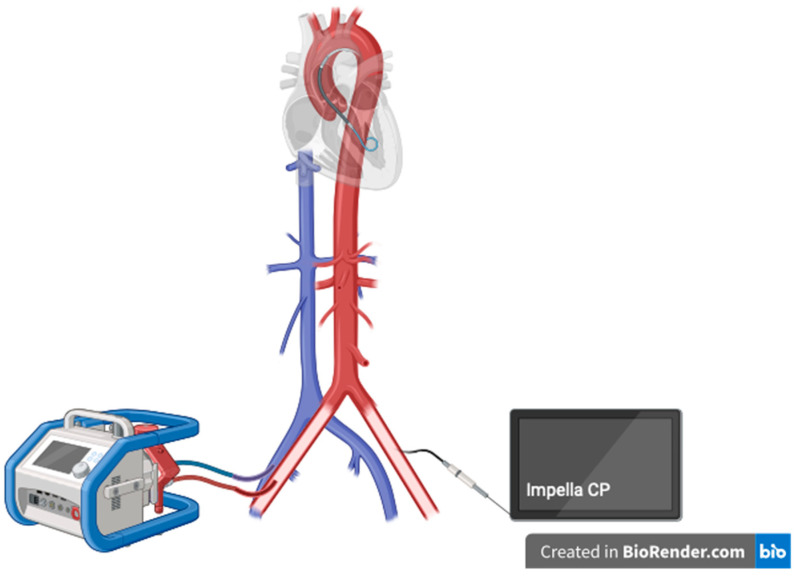
ECPELLA configuration for LV unloading.

LV overload after VA ECMO implantation puts myocardial recovery in danger. Unloading of the LV leads to the reduction in the LV end-diastolic pressure, reduction in the pressure in the left atrium, and the decrease in the LV thrombus formation risk. To conclude, better conditions for myocardial recovery, with comfortable filling pressures and a better oxygen delivery/demand ratio, are achieved [20]. Currently, a growing evidence on possible lower mortality associated with the use of Impella device as a modality for the LV unloading in ECMO patients evolves. The combination of VA ECMO and Impella is usually labeled as ECPELLA or ECMELLA, which is shown in Scheme 2.

Drawbacks of using Impella are increased risk of bleeding complications, hemolysis, and abdominal compartment syndrome [20]. Notably, the use of ECPELLA brings a higher risk of need for renal replacement therapy (RRT) in comparison with VA ECMO alone [20]. The rational consequence of the ECPELLA configuration is the higher risk of Harlequin syndrome, whereas adding another venous cannula via the *internal jugular vein* (for the return of oxygenated blood) and transformation to V-AV ECMO + Impella should be a solution to the issue [21].

Impella family devices are contraindicated for patients with LV thrombosis, mechanical aortic valve prosthesis, moderate-to-severe aortic valve disease, and severe peripheral artery disease (due to the risk of limb ischemia and/or technical issues with implantation). The above-mentioned list of limitations is even more bounding in critically ill patients due to the higher frequency of these pathological conditions.

## 4. Current Evidence for Using ECPELLA

The pioneers of the new approach—Pappalardo et al.—published in 2017, the first worldwide, a retrospective observational study on 157 patients. The group with ECPELLA showed significantly lower (47% vs. 80%, *p <* 0.001) in-hospital mortality than patients with VA ECMO only [22]. A meta-analysis performed by Silvestri et al. in 2020 included 448 patients in total, showing results with the same trend, the lower mortality of the ECPELLA group (52.6% vs. 63.6%, *p* < 0.01) against VA ECMO only [23].

Recent meta-analysis published by Fiorelli and Panoulas in 2021 included 972 patients, which were divided into the ECPELLA and ECMO-only groups. After excluding studies without a homogenous comparator group, the combination of Impella with VA ECMO was still associated with lower mortality risk (RR: 0.85; 95% CI: 0.75, 0.97; *p* = 0.01). On the other hand, hemolysis (RR: 1.70; 95% CI: 1.35, 2.15; *p* < 0.00001) and RRT (RR: 1.86; 95% CI: 1.07, 3.21; *p* = 0.03) occurred at a higher rate in the group of patients with ECPELLA. No significant difference was observed in terms of major bleeding complications between the groups (RR: 1.37; 95% CI: 0.88, 2.13; *p* = 0.16) and cerebrovascular accidents (RR: 0.91; 95% CI: 0.61, 1.38; *p* = 0.66) [24].

A huge retrospective study performed based on ELSO registry by Grandin et al. in 2022 showed an even wider image of LV unloading. The big data from ELSO registry contained 12,734 patients with VA ECMO (from years 2010 to 2019), of which 26.7% were upgraded with mechanical unloading device—IABP (in 82.9%) or Impella (in 17.1%). Patients who were finally treated with VA ECMO + IABP/ECPELLA were, before VA ECMO cannulation, in more serious condition than those who did not require LV unloading. The LV-unloaded patients required >2 vasopressors more frequently (41.7% vs. 27.2%) and had respiratory (21.1% vs. 15.9%), renal (24.6% vs. 15.8%), or liver failure (4.4% vs. 3.1%) (all *p<* 0.001) before the VA ECMO implantation also more often. However, importantly, in these basically more severely ill patients, a lower in-hospital mortality rate was observed (56.6% vs. 59.3%, *p* = 0.006), which remained lower in a multivariable modeling (adjusted OR (aOR): 0.84; 95% CI: 0.77–0.92; *p <* 0.001). What was not significant in this retrospective analysis is a mortality difference in the ECPELLA vs. VA ECMO + IABP group (aOR: 0.80; 95% CI: 0.64–1.01; *p* = 0.06) [25].

A large meta-analysis by Russo et al. (2019) included 3997 patients, of which 42% underwent LV unloading on VA ECMO. The mortality in the group of patients with LV unloading was again significantly lower (54%) in comparison with the group of patients with VA ECMO only (65%). Surprisingly, in this research, 91.7% of cases were unloaded with IABP vs. 5.5% with Impella [26]. Despite this, the mortality was lower.

A comprehensive insight was brought into the topic by Schrage et al. (2020). This multi-center international cohort study (686 patients) confirmed the other results. The mortality of ECPELLA group subjects was 58.3% (95% CI, 51.6–64.1%) vs. 65.7% (95% CI, 59.2–71.2%) in ECMO-only subjects. Additionally, the analysis of subgroups was performed to explore the role of right timing of LV unloading and initiation. The data showed that early LV unloading was associated with significantly lower 30-day mortality (HR, 0.76 (95% CI, 0.60–0.97); *p* = 0.03), but delayed LV unloading (defined as Impella implantation >2 h after VA ECMO implantation) was not associated with better mortality outcome (HR, 0.77 (95% CI, 0.51–1.16); *p* = 0.22) [27]. Another light has been recently brought into the discussion by the same team of Schrage et al. (2023). The data of 421 subjects with cardiogenic shock treated with VA ECMO and active LV unloading at 18 centers were analyzed. Early active unloading of the LV was initiated in 73.6% of the patients. The results showed that early unloading of the LV was associated with a lower 30-day mortality risk (HR: 0.64; 95% CI: 0.46–0.88). The same group of patients was characterized with a higher chance for successful weaning from ventilation (OR: 2.17; 95% CI: 1.19–3.93) [28]. Thus, the discussion about the ideal timing of LV unloading initiation should be underlined.

## 5. Conclusions

The ECPELLA approach seems to be a promising strategy that may bring the improvement of CS mortality rates. The series of presented trials and meta-analyses clearly showed the potential benefits of this strategy. However, the ongoing research has brought a series of new questions, such as whether Impella itself is the only right unloading modality, or any other approach to unload the LV would be beneficial in the same way. Furthermore, the discussion about the right timing of the LV unloading initiation was opened. On the other hand, ECPELLA requires additional arterial access, and its association with the increased rate in bleeding complications and hemolysis is also clearly based on the significant evidence.

As already mentioned, it is clear that this kind of research is connected to multiple challenges including not only statistical and medical but also ethical and legal issues associated with designing such trials. Considering a low absolute number of patients with CS shock indicated for VA ECMO treatment, followed by a thorough selection of subjects, it is clear that a single-center trial would not be able to collect sufficient data. Thus, experienced international teams should be those who might be eligible to design and manage multi-center trials without significant mistakes in the architecture of the study.

From a “bedside-clinician” point of view, understanding of VA ECMO-driven circulation pathophysiology is crucial for further hemodynamic assessment of possible LV overload. Analysis of multiple measurements, such as loss of arterial line curve pulsatility, PAC values, echocardiographic evaluation of the aortic valve remaining closed, LV dilation, and progressive hypoxemia (altogether with lung ultrasound and X-ray finding), may lead to the decision to initiate LV unloading.

## Figures and Tables

**Table 1:** Different types of mechanical circulatory/cardiac support devices (based on Atti et al. [11]; AI = aortic insufficiency, PAD = peripheral artery disease, AAA = abdominal aortic aneurysm).

	IABP	Impella Family	TandemHeart	VA ECMO
**Pump Mechanism**	Pneumatic	Axial flow	Centrifugal	Centrifugal
**LV unloading**	+	++	++	Possible LV overload
**Advantages**	Bedside insertion, economy	Quality of unloading	Addition of pulmonary support	Bedside insertion and addition of pulmonary support
**Disadvantages**	Minimal hemodynamic support	Significant risk of hemolysis	Immobilization	No LV/RV support
**Contraindications**	AAA, severe AI	LV thrombus, mechanical aortic valve, severe PAD	Ventricular septal defect, severe AI	Severe PAD, severe AI

## Data Availability

No new data in this review.

## References

[1] ECMO Registry of the Extracorporeal Life Support Organization (ELSO), Ann Arbor, Michigan, June 2023. https://www.elso.org/registry.aspx.

[2] Ostadal P., Rokyta R., Karasek J., Kruger A., Vondrakova D., Janotka M., Naar J., Smalcova J., Hubatova M., Hromadka M. (2023). Extracorporeal Membrane Oxygenation in the Therapy of Cardiogenic Shock: Results of the ECMO-CS Randomized Clinical Trial. Circulation.

[3] Thiele H., Zeymer U., Akin I., Behnes M., Rassaf T., Mahabadi A.A., Lehmann R., Eitel I., Graf T., Seidler T. (2023). Extracorporeal Life Support in Infarct-Related Cardiogenic Shock. N. Engl. J. Med..

[4] Zeymer U., Freund A., Hochadel M., Ostadal P., Belohlavek J., Rokyta R., Massberg S., Brunner S., Lüsebrink E., Flather M. (2023). Venoarterial extracorporeal membrane oxygenation in patients with infarct-related cardiogenic shock: An individual patient data meta-analysis of randomised trials. Lancet.

[5] Hill J.D., O’Brien T.G., Murray J.J., Dontigny L., Bramson M.L., Osborn J.J., Gerbode F. (1972). Prolonged Extracorporeal Oxygenation for Acute Post-Traumatic Respiratory Failure (Shock-Lung Syndrome). N. Engl. J. Med..

[6] Stretch R., Sauer C.M., Yuh D.D., Bonde P. (2014). National Trends in the Utilization of Short-Term Mechanical Circulatory Support: Incidence, Outcomes, and Cost Analysis. J. Am. Coll. Cardiol..

[7] Cavarocchi N.C. (2017). Introduction to Extracorporeal Membrane Oxygenation. Crit. Care Clin..

[8] Rao P., Khalpey Z., Smith R., Burkhoff D., Kociol R.D., Mehmood M., Alhussein M., Moayedi Y., Posada J.D., Ross H. (2018). Venoarterial Extracorporeal Membrane Oxygenation for Cardiogenic Shock and Cardiac Arrest. Circ. Heart Fail..

[9] de Waha S., Fuernau G., Eitel I., Desch S., Thiele H. (2016). Long-term prognosis after extracorporeal life support in refractory cardiogenic shock—Results from a real-world cohort. EuroIntervention.

[10] Pavasini R., Cirillo C., Campo G., Nobre Menezes M., Biscaglia S., Tonet E., Ferrari R., Patel B.V., Price S. (2017). Extracorporeal Circulatory Support in Acute Coronary Syndromes: A Systematic Review and Meta-Analysis. Crit. Care Med..

[11] Atti V., Narayanan M.A., Patel B., Balla S., Siddique A., Lundgren S., Velagapudi P. (2022). A Comprehensive Review of Mechanical Circulatory Support Devices. Heart Int..

[12] El Sibai R., Bachir R., El Sayed M. (2018). ECMO use and mortality in adult patients with cardiogenic shock: A retrospective observational study in U.S. hospitals. BMC Emerg. Med..

[13] Belohlavek J., Hunziker P., Donker D.W. (2021). Left ventricular unloading and the role of ECpella. Eur. Heart J. Suppl..

[14] Burkhoff D., Sayer G., Doshi D., Uriel N. (2015). Hemodynamics of Mechanical Circulatory Support. J. Am. Coll. Cardiol..

[15] Rupprecht L., Lunz D., Philipp A., Lubnow M., Schmid C. (2015). Pitfalls in percutaneous ECMO cannulation. Heart Lung Vessel..

[16] Meani P., Gelsomino S., Natour E., Johnson D.M., La Rocca H.-P.B., Pappalardo F., Bidar E., Makhoul M., Raffa G., Heuts S. (2017). Modalities and Effects of Left Ventricle Unloading on Extracorporeal Life support: A Review of the Current Literature. Eur. J. Heart Fail..

[17] Hogue C.W., Levine A., Hudson A., Lewis C. (2021). Clinical Applications of Near-infrared Spectroscopy Monitoring in Cardiovascular Surgery. Anesthesiology.

[18] Al-Fares A.A., Randhawa V.K., Englesakis M., McDonald M.A., Nagpal A.D., Estep J.D., Soltesz E.G., Fan E. (2019). Optimal Strategy and Timing of Left Ventricular Venting During Veno-Arterial Extracorporeal Life Support for Adults in Cardiogenic Shock: A Systematic Review and Meta-Analysis. Circ. Heart Fail..

[19] Glazier J.J., Kaki A. (2019). The Impella Device: Historical Background, Clinical Applications and Future Directions. Int. J. Angiol..

[20] Meani P., Lorusso R., Pappalardo F. (2022). ECPella: Concept, Physiology and Clinical Applications. J. Cardiothorac. Vasc. Anesth..

[21] Giunta M., Recchia E.G., Capuano P., Toscano A., Attisani M., Rinaldi M., Brazzi L. (2023). Management of harlequin syndrome under ECPELLA support: A report of two cases and a proposed approach. Ann. Card. Anaesth..

[22] Pappalardo F., Schulte C., Pieri M., Schrage B., Contri R., Soeffker G., Greco T., Lembo R., Müllerleile K., Colombo A. (2017). Concomitant implantation of Impella^®^ on top of veno-arterial extracorporeal membrane oxygenation may improve survival of patients with cardiogenic shock. Eur. J. Heart Fail..

[23] Grajeda Silvestri E.R., Pino J.E., Donath E., Torres P., Chait R., Ghumman W. (2020). Impella to unload the left ventricle in patients undergoing venoarterial extracorporeal membrane oxygenation for cardiogenic shock: A systematic review and meta-analysis. J. Card. Surg..

[24] Fiorelli F., Panoulas V. (2021). Impella as unloading strategy during VA-ECMO: Systematic review and meta-analysis. Rev. Cardiovasc. Med..

[25] Grandin E.W., Nunez J.I., Willar B., Kennedy K., Rycus P., Tonna J.E., Kapur N.K., Shaefi S., Garan A.R. (2022). Mechanical Left Ventricular Unloading in Patients Undergoing Venoarterial Extracorporeal Membrane Oxygenation. J. Am. Coll. Cardiol..

[26] Russo J.J., Aleksova N., Pitcher I., Couture E., Parlow S., Faraz M., Visintini S., Simard T., Di Santo P., Mathew R. (2019). Left Ventricular Unloading During Extracorporeal Membrane Oxygenation in Patients With Cardiogenic Shock. J. Am. Coll. Cardiol..

[27] Schrage B., Becher P.M., Bernhardt A., Bezerra H., Blankenberg S., Brunner S., Colson P., Cudemus Deseda G., Dabboura S., Eckner D. (2020). Left Ventricular Unloading Is Associated With Lower Mortality in Patients With Cardiogenic Shock Treated With Venoarterial Extracorporeal Membrane Oxygenation: Results From an International, Multicenter Cohort Study. Circulation.

[28] Schrage B., Sundermeyer J., Blankenberg S., Colson P., Eckner D., Eden M., Eitel I., Frank D., Frey N., Graf T. (2023). Timing of Active Left Ventricular Unloading in Patients on Venoarterial Extracorporeal Membrane Oxygenation Therapy. JAC Heart Fail..

